# Coronary Microvascular Dysfunction: Insights on Prognosis and Future Perspectives

**DOI:** 10.31083/RCM25757

**Published:** 2025-01-20

**Authors:** Filippo Luca Gurgoglione, Giorgio Benatti, Andrea Denegri, Davide Donelli, Marco Covani, Mattia De Gregorio, Gabriella Dallaglio, Rebecca Navacchi, Giampaolo Niccoli

**Affiliations:** ^1^Division of Cardiology, University of Parma, Parma University Hospital, 14 - 43126 Parma, Italy; ^2^Division of Cardiology, Parma University Hospital, 14 - 43126 Parma, Italy

**Keywords:** coronary microvascular dysfunction, major adverse cardiovascular events, heart failure with preserved ejection fraction, quality of life

## Abstract

Coronary microvascular dysfunction (CMD) comprises a wide spectrum of structural and/or functional abnormalities of coronary microcirculation that can lead to myocardial ischemia. Emerging evidence has indicated that CMD is a relevant cause of morbidity and mortality and is associated with a high risk of major adverse cardiovascular events (MACEs) and heart failure with preserved ejection fraction as well as poor quality of life. This review aims to elucidate briefly the pathogenesis and diagnostic modalities of CMD and to shed light on contemporary evidence on the prognostic impact of CMD. Finally, we will provide an overview of novel emerging therapeutic strategies for CMD.

## 1. Introduction

Coronary microvascular dysfunction (CMD) refers to the inability of coronary microcirculation to dilate in response to increased myocardial oxygen demand due to a combination of structural and functional alterations [1]. CMD is an increasingly recognized mechanism of myocardial ischemia and a major determinant of ischemia with non-obstructive coronary artery (INOCA) disease [2, 3, 4] and chronic coronary syndrome (CCS) [5].

CCS encompasses a wide spectrum of clinical presentations characterized by an imbalance between myocardial oxygen supply and demand, ultimately resulting in myocardial ischemia. The underlying pathophysiology is complex, involving both structural and functional alterations in the coronary arteries and/or microcirculation [5].

Obstructive coronary artery disease (CAD) is the most common and well-known cause of myocardial ischemia. Moreover, CAD is characterized by the progressive accumulation of lipids and inflammatory cells within the coronary arteries, forming atherosclerotic lesions covered by a fibrous cap. Further, this gradual narrowing of the coronary arterial lumen can lead to myocardial hypoperfusion, particularly during physical exertion and emotional or other stresses [5].

Coronary vasomotor disorders represent the primary functional cause of CCS and are characterized by transient hypercontraction of coronary vascular smooth muscle cells. Endothelial dysfunction and increased vascular smooth muscle cell reactivity underlie these disorders [2, 3]. Coronary artery spasms may occur at the epicardial level, diagnosed when a ≥90% reduction in epicardial coronary diameter is observed alongside the reproduction of the patient’s symptoms and ischemic changes on an electrocardiogram (ECG). Alternatively, spasms can affect the microvascular circulation, defined by the presence of typical angina and ischemic ECG changes without significant epicardial coronary constriction [2]. Indeed, invasive provocative testing with intracoronary acetylcholine administration is the gold standard for diagnosing coronary vasomotor disorders, providing important therapeutic and prognostic insight [6, 7].

Interestingly, a recent meta-analysis involving 14,427 patients demonstrated that 23% of individuals with INOCA presented with both CMD and vasomotor disorders [8]. Notably, these functional alterations can also coexist with obstructive CAD. These findings highlight the importance of a comprehensive functional assessment of the coronary vasculature to identify the precise mechanisms underlying CCS and to facilitate the development of tailored medical therapies [9].

The condition involving chest pain in the absence of obstructive CAD has been recognized since the 1970s and was initially termed “cardiac syndrome X” [10]; however, the term CMD has recently been introduced. This condition is primarily associated with microvascular dysfunction, endothelial impairment, and heightened pain sensitivity. Notably, it is more prevalent in women than men [11].

While initially considered a neglected condition, the advent of non-invasive and invasive functional tests has exponentially broadened the ability to recognize CMD and delineate related clinical and biochemical features [3, 8].

Convincing evidence has suggested that CMD is not as benign as previously thought. Indeed, CMD confers a high risk of major adverse cardiovascular events (MACEs) [12] and heart failure with preserved ejection fraction (HFpEF) [13], while patients with CMD also often experience a poor quality of life (QOL) [14].

This review aims to provide a brief overview of the pathogenesis and invasive diagnosis of CMD and to elucidate contemporary evidence on the prognostic impact of CMD. Finally, we will discuss novel potential therapeutic strategies for personalized CMD treatments.

## 2. Epidemiology

Although epicardial CAD is mainly responsible for myocardial ischemia, up to half of the subjects undergoing invasive coronary angiography for symptoms or non-invasive evidence of myocardial ischemia have non-obstructive CAD [5]. The spectrum of conditions underpinning myocardial ischemia and its symptomatic manifestation in the absence of obstructive CAD is generally referred to by the acronym INOCA [3]; meanwhile, a key mechanism underlying INOCA is CMD, characterized by a reduced coronary flow reserve (CFR) [3].

CMD can be classified into four categories based on clinical presentation: (1) CMD in the absence of myocardial disease and obstructive CAD; (2) CMD in myocardial disease; (3) CMD in obstructive CAD; (4) iatrogenic CMD [15].

Studies with adenosine and acetylcholine as vasodilator agents suggested that CMD was present in 60% of patients with non-obstructive CAD [16, 17, 18]. A further study found CMD in up to 40% of cases, although a transthoracic Doppler echocardiography seems to be characterized by low accuracy [19]. Conversely, positron emission tomography (PET) has shown the presence of CMD in 60% of cases, with no significant gender differences [20].

## 3. Pathophysiology

CMD has been shown to primarily arise from abnormalities in the coronary microvascular circulation [2, 3]. The coronary microcirculation comprises a network of vessels with a diameter of <500 µm, encompassing pre-arterioles, arterioles, and capillaries. This network is the main contributor to the total resistance of the coronary vasculature in the absence of significant obstructive disease of large epicardial vessels. Under physiological circumstances, this network can modulate an increase in myocardial blood flow of up to five times the basal one in response to increased metabolic demands. CMD also shares common risk factors with atherosclerotic disease, including advancing age, hypertension, insulin resistance, obesity, smoking, and hyperlipidemia [21].

From a pathophysiological standpoint, CMD results from a variable combination of microcirculatory functional and structural abnormalities [15] that can altogether lead to reduced CFR, augmented microvascular resistance, and paradoxical arteriolar vasoconstriction, which can be provoked during invasive provocative tests, such as acetylcholine infusion.

Several hypotheses have been proposed to explain the multifaceted mechanisms underlying CMD. Endothelial dysfunction is considered a key mediator of functional dysregulation affecting the microvasculature. Further, endothelial dysfunction involves a maladaptive shift towards a net vasoconstrictive state, promoted by a reduction in the bioavailability of vasodilatory agents such as prostaglandins, nitric oxide (NO), and endothelium-derived hyperpolarizing factors and through an increased release of constrictive agents such as endothelin-1 (ET-1), thromboxane A2, prostaglandin H2, and reactive oxygen species (ROS) [22, 23].

In addition, endothelium-independent mechanisms, including impaired relaxation of vascular smooth muscle cells jointly with enhanced RhoA/Rho kinase activity and increased vasoconstrictive mediators, contribute to the pathogenesis of CMD [24] and microvascular spasms [25].

Inflammation and neurohormonal disarrangements also play a pivotal role. A pro-inflammatory state promotes oxidative stress and endothelial dysfunction through a complex interplay involving interleukin-6, tissue necrosis factor α, chemokines, adipokines, and ROS, as proven by the association between CMD and higher serum C-reactive protein levels [26]. Additionally, sympathetic dysfunction and renin–angiotensin–aldosterone system activation can promote an abnormal vasoconstrictive response mediated by α-adrenergic receptors and angiotensin II [27].

Lastly, structural changes also contribute to the pathogenesis of CMD. These include luminal narrowing of small resistance arterioles with hypertrophic inward remodeling, increased stiffness with perivascular fibrosis, and capillary rarefaction [28].

## 4. Invasive Diagnosis

The limited resolution of coronary angiography has spurred the implementation of invasive techniques that can test coronary microvascular status selectively. A reduced CFR, the hallmark of CMD [3, 4], provides a unique opportunity to evaluate both epicardial and microvascular compartments [12]. Two strategies are currently available to measure CFR. The Doppler flow velocity method utilizes a Doppler wire (ComboWire XT or FloWire, Philips Volcano Corporation, SAN DIEGO, CA, USA) to measure coronary flow velocity (CFV) at rest and following a hyperemic stimulus. Adenosine is the most used vasodilator agent in this context, even though intracoronary papaverine is gaining popularity. The hyperemic to the resting CFV ratio offers a reliable quantification of CFR with pathological values <2.5 [29]. Thermodilution is an alternative method that requires a dedicated pressure wire equipped with three sensors: proximal and distal temperature and distal pressure (PressureWire XTM, Abbott Vascular, SANTA CLARA, CA, USA). This strategy (bolus thermodilution) leverages three saline injections at room temperature during rest and during maximal hyperemia to calculate the mean transit time (Tmn); the ratio of resting to the hyperemic Tmn is a reliable method to estimate CFR with normal values >2 [30]. Recently, a novel strategy that leverages continuous intracoronary thermodilution through a dedicated catheter has been implemented with putative advantages regarding precise coronary flow measurements and absolute microcirculatory resistance [31].

Invasive function testing features the unique potential to categorize CMD into structural and functional through the assessment of microvascular resistance [32]. Two indices are available in clinical practice: The index of microcirculatory resistance (IMR), which is a thermodilution-based index, defined as the product of distal coronary pressure and Tmn during maximal hyperemia, with normal values <25 [33]; the hyperemic microvascular resistance (hMR), which is an alternative method for testing microvascular dilatory function using a dual sensor Doppler flow and pressure wire system. Overall, hMR values ≥2.5 are suggestive of CMD [34].

## 5. Prognosis

**Fig. 1.**
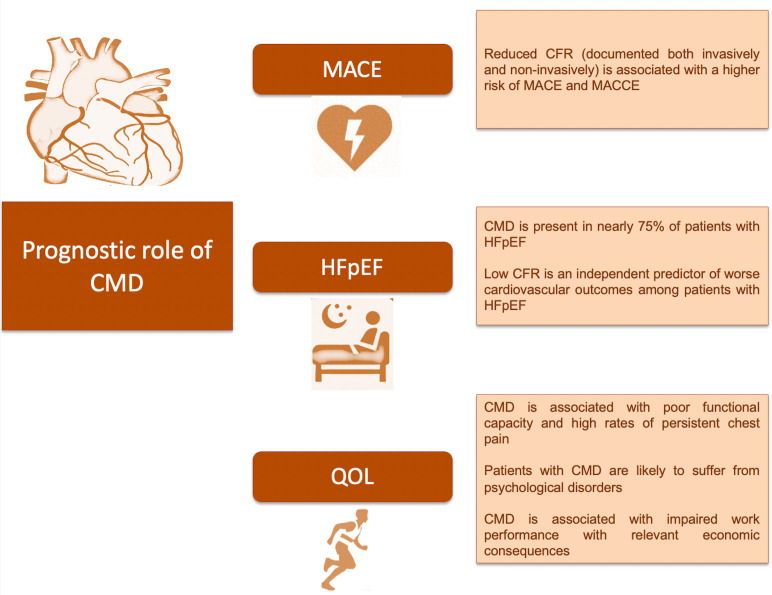
**Overview of possible complications associated with CMD**. Abbreviations: CFR, coronary flow reserve; CMD, coronary microvascular dysfunction; HFpEF, heart failure with preserved ejection fraction; MACE, major adverse cardiovascular event; MACCE, major adverse cardiovascular and cerebrovascular event; QOL, quality of life.

A CMD diagnosis is associated with a wide spectrum of complications, including a high risk of MACEs and HFpEF and poor QOL, compared to healthy individuals, and that includes approximations of the complications associated with patients with obstructive CAD (Fig. 1).

### 5.1 MACEs

Patients presenting with angina and not obstructive CAD are generally reassured that their symptoms are noncardiac. However, in the presence of CMD, several studies have demonstrated that these patients are at increased risk of MACEs [12, 35]. Suwaidi *et al*. [36] showed that severe endothelial dysfunction, assessed through an impaired response to acetylcholine stimulus, is associated with increased cardiac events at a median follow-up of 28 months, confirmed in subsequent studies with longer follow-ups [37, 38].

The Women’s Ischemia Syndrome Evaluation (WISE) study demonstrated that female subjects with symptoms and signs suggestive of ischemia but without obstructive CAD present a 5-fold increased risk of MACEs compared with asymptomatic women with no history of heart disease [39]; however, recent evidence has refuted these findings, albeit an increased risk of MACEs (8.6% *vs*. 3.5%, *p* < 0.001) was observed among symptoms and cardiovascular risk factors [20]. Another recent study using intracoronary CFR demonstrated that impaired CFR ≤2.0 was associated with a 3-fold increased risk of vessel-oriented composite outcome (*i.e.*, vessel-related cardiac death, vessel-specific myocardial infarction (MI), and vessel-specific revascularization), among 519 patients with CAD who did not undergo revascularization (adjusted hazard ratio (adj. HR) 3.2, 95%, confidence interval (CI) 1.7–6.0, *p* < 0.001) [40].

Asymptomatic diabetic patients (n = 101) with impaired CFR in an adenosine stress echocardiography presented an increased risk of MACEs (adj. HR 12.9, 95% CI 3.9–43.2, *p* < 0.001) [41]; similar results were detected in 317 subjects referred to myocardial perfusion scintigraphy for suspected myocardial ischemia (adj. HR 3.0, 95% CI 1.5–6.0, *p* = 0.002) [42]. The prognostic value of coronary vasodilator function was analyzed in 451 people with diabetes without CAD and normal perfusion who presented lower event-free survival compared to nondiabetic patients with impaired myocardial flow reserve (annualized event rate, 1.4% *vs*. 0.3%, *p* < 0.001) [43].

In patients with suspected myocardial ischemia (n = 229), PET-derived abnormal CFR was associated with increased risk of MACEs (adj. HR 1.60, 95% CI 1.00–2.57, *p* < 0.05; 45.1% *vs*. 23.6%, *p* < 0.05) and cardiovascular (CV) death (adj. HR 2.86, 95% CI 1.24–6.59, *p* < 0.001; 20.6% *vs*. 6.3%, *p* < 0.001) [44, 45].

Decreased CFR in a stress echocardiogram has been associated with markedly increased risk in women (adj. HR 16.5, 95% CI 7.2–37.9, *p* < 0.001) and men (adj. HR 6.2, 95% CI 3.4–11.3, *p* < 0.001) with chest pain and a normal result on a dipyridamole stress echocardiography [46]. Subsequent analyses performed by the same group revealed, among 4313 patients with known (n = 1547) or suspected (n = 2766) CAD, that impaired CFR is associated with increased mortality (adj. HR 3.31, 95% CI 2.29–4.78, *p* < 0.001) and confer additional prognostic value on top of wall motion abnormalities [47, 48].

In the absence of obstructive coronary stenoses, abnormal non-invasive stress tests in patients with stable CAD may indicate INOCA; however, this absence does not identify patients with a higher risk of long-term cardiovascular events. A recent analysis was conducted on 297 patients with a positive non-invasive test and nonobstructive coronary stenoses (fractional flow reserve ≥0.80) from the international multicenter registry of intracoronary physiologic assessment (ILIAS (Inclusive Invasive Physiological Assessment in Angina Syndromes) registry, N = 2322), and integrated intracoronary physiologic assessment information to re-classify patients in different subgroups found up to a 4-fold difference in long-term cardiovascular events (adj. HR 2.88; 95% CI, 1.52–7.19; *p* = 0.024 to adj. HR, 4.00; 95% CI, 1.41–11.35; *p* = 0.009) [49].

The prognostic value of IMR was tested in this context. Liu *et al*. [50] conducted a study including 151 consecutive patients with chest pain and non-obstructive CAD who largely presented CMD (61.6%). Patients with CMD, assessed through an impaired CFR (<2.5), showed an increased risk of MACEs (HR 3.1, 95% CI 1.2–7.9, *p* = 0.017) than non-CMD patients, with CMD found as an independent predictor of MACEs [50].

The coronary microcirculatory function has recently been investigated with microvascular resistance reserve (MMR) in 547 consecutive patients undergoing systematic echocardiography and invasive physiological assessment for suspected CAD. A study demonstrated that an impaired MMR ≤3.0 was associated with a composite of cardiovascular death, MI, repeat revascularization, and admission for heart failure (HF) in patients with CCS, irrespective of significant epicardial coronary artery stenosis (HR 1.23 per 1 U decrease; 95% CI: 1.12–1.36; *p* < 0.001) [51].

Finally, patients with CMD experienced a nearly 4-fold higher risk of overall mortality compared to healthy controls [52]: this finding suggests that reduced CFR might be a marker of systemic endothelial dysfunction [53] (Table 1, Ref. [20, 35, 36, 37, 38, 40, 41, 42, 44, 45, 46, 47, 48, 49, 50]).

**Table 1.** **Non-invasive and invasive tests for CMD evaluation and related crude outcome**.

First author, year [Ref]	Sample size	Measure modality	Type of outcome	Rate of outcome
Non-invasive tests				
Herzog, 2009 [44]	229	Adenosine 13-N ammonia-PET	MACEs	45.1% *vs*. 23.6%
Cortigiani, 2010 [46]	1660	Stress echocardiography	MACEs	27.0% *vs*. 2.0%*
		LAD CFR		42.0% *vs*. 8.0%**
Ziadi 2011 [45]	414	Dypiridamole rubidium82-PET	MACEs	24.0% *vs*. 9.0%^#^
Cortigiani, 2012 [47]	4313	CFR on LAD	Death	39.0% *vs*. 7.0%
Murthy, 2014 [20]	1218	Vasodilator rubidium82-PET	MACEs	8.6% *vs*. 3.5%
Dikic, 2015 [41]	200	Adenosine stress echocardiography	MACEs	18.8% *vs*. 5.1%
Gan, 2017 [42]	371	Adenosine stress echocardiography	MACEs	36.8% *vs*. 10.8%
Cortigiani, 2018 [48]	375	CFVR and LVCR	MACEs	63.0% *vs*. 10%^+^
Invasive tests				
Suwaidi, 2000 [36]	157	CBF	MACEs	14.0% *vs*. 0.0%
Schächinger, 2000 [38]	147	CBF	MACEs	11.0%***
Britten, 2004 [37]	120	CFR	MACEs	18.0% *vs*. 5.0%
Marks, 2004 [35]	168	CFR	Death	20.0% *vs*. 7.0%
Lee, 2018 [40]	631	CFR	VOCO	11.2% *vs*. 3.7%
Boerhout, 2022 [49]	1102	CFR + MR	MACEs	11.7% *vs*. 5.5%
Liu, 2023 [50]	151	calMR	MACCEs	40.9% *vs*. 13.8%

Abbreviations: CMD, coronary microvascular dysfunction; VOCO, vessel-oriented clinical outcomes; calMR, coronary angiography-derived index of microvascular resistance; CBF, coronary blood flow; CFR, coronary flow reserve; CFVR, coronary flow velocity reserve; LAD, left anterior descending artery; LVCR, left ventricular contractile reserve; MACEs, major adverse cardiovascular events; MACCEs, major adverse cardiovascular and cerebrovascular events; MR, microvascular resistance; PET, positron emission tomography; *women; **men; ***overall population; ^#,+^intergroups.

### 5.2 HFpEF

HFpEF is a clinical syndrome consisting of typical symptoms and signs of HF in the presence of cardiac structural and/or functional abnormalities, which generally lead to raised left ventricular filling pressures with preserved systolic function [54]. In recent years, alongside a growing understanding of this condition, a new paradigm has emerged that recognizes an important link with microcirculatory dysfunction. Several studies have demonstrated a consistently high prevalence of CMD among patients with HFpEF [13, 55, 56, 57], although the mutual causative interplay of these two entities is still a matter of uncertainty. Interestingly, HFpEF and CMD share similar risk factors (e.g., hypertension, advanced age, female sex, chronic kidney disease, diabetes mellitus, obesity) [58, 59], and the common denominator between these two conditions is believed to be represented by endothelial dysfunction promoted by a comorbidity-driven systemic pro-inflammatory and pro-oxidative state [60]. While endothelial dysfunction may exist in both heart failure with reduced and preserved ejection fractions, mounting evidence points toward its primary role in the pathogenesis of HFpEF. Indeed, cardiac endothelium directly affects the contractility and relaxation of underlying myocardial cells [61]. Consequently, inflamed microvascular endothelium can contribute to diastolic dysfunction, the hallmark of HFpEF, through reduced NO-bioavailability and the release of transforming growth factor-β, which promote impaired myocyte relaxation, myofibroblast proliferation, interstitial fibrosis, and vascular rarefaction, as shown in animal and human models [62, 63, 64].

The presence of CMD is also an important prognostic factor in patients affected by HFpEF [65, 66, 67, 68].

The PROMIS-HFpEF (PRevalence Of MIcrovascular dySfunction in Heart Failure with Preserved Ejection Fraction), the largest prospective study to date, showed a high prevalence (75%) of CMD (defined as CFR <2.5 in an adenosine stress transthoracic Doppler echocardiography) among 202 patients with HFpEF. The PROMIS-HFpEF study also demonstrated an independent association between CMD and systemic endothelial dysfunction and with markers of heart failure severity, such as natriuretic peptides and indices of right heart systolic dysfunction [69]. In a pre-specified exploratory analysis, the presence of CMD was demonstrated to be independently associated with a significantly higher risk of cardiovascular and heart failure adverse events at the 1-year follow-up [70].

The majority of data regarding the prognostic role of CMD in patients with HFpEF comes from cardiac magnetic resonance (CMR) imaging studies. Kato *et al*. [71], in a retrospective study including 163 patients with HFpEF undergoing vasodilator stress CMR, demonstrated that CFR was significantly lower in HFpEF patients with adverse events compared with those without. Furthermore, Kaplan–Meier analysis showed that at a median follow-up of 4.1 years, rates of all-cause death and heart failure hospitalizations were significantly higher in HFpEF patients with CFR <2.0 compared with those in HFpEF patients with CFR ≥2.0 (*p* < 0.001) [71]. Accordingly, in the DIAMOND-HFpEF study, Arnold *et al*. [72] showed that among 101 patients with HFpEF low (<2.0), CMR-derived myocardial perfusion reserve was independently associated with poorer cardiovascular outcomes at a median follow-up of 3.1 years.

In conclusion, growing evidence strengthens the implementation of CMD as a useful prognostic marker for HFpEF. Moreover, given the frequent association between CMD and HFpEF, targeting endothelial dysfunction appears to be a promising therapeutic strategy for novel interventions. Nonetheless, it must be emphasized that despite the fascinating underlying pathophysiological hypothesis, more research is needed to clarify a cause-and-effect relationship between these two entities (Table 2, Ref. [13, 55, 56, 57, 65, 66, 68, 69, 70, 71, 72]).

**Table 2.** **Main features of studies on coronary microvascular dysfunction and heart failure with preserved ejection fraction**.

First author, year [Ref]	Population, comparison	CMD assessment method, CMD definition	Timeframe	Key findings
Sucato, 2015 [55]	n = 155 HFpEF, n = 131 non-HFpEF	Invasive coronary angiography, NA	NA	Significantly lower TFC and MBG of the three coronary arteries in HFpEF *vs*. non-HFpEF patients
Taqueti, 2018 [13]	n = 201 non-HFpEF, NA	Rb-82 PET-derived CFR, CFR <2.0	Median follow-up of 4.1 years	Using a multivariate analysis, CMD was independently predictive of CV death, MI, and/or HFpEF hospitalization
Shah, 2018 [69]	n = 202 HFpEF, NA	TTE-derived CFR, CFR <2.5	NA	Prevalence of CMD of 75% (95% CI: 69–81%) Poorer CFR was independently associated with markers of endothelial dysfunction and HF severity
Dryer, 2018 [56]	n = 30 HFpEF, n = 14 non-HFpEF	CFR and IMR through coronary pressure wire, CFR ≤2.0, IMR ≥23	NA	Significantly lower mean CFR (2.55 ± 1.60 *vs*. 3.84 ± 1.89, *p* = 0.024) and higher mean IMR (26.7 ± 10.3 *vs*. 19.7 ± 9.7, *p* = 0.037) in HFpEF compared with controls
Yang, 2020 [65]	n = 162 HFpEF, NA	Endothelium-dependent: increase in CBF after acetylcholine administration Endothelium-independent: CFR through coronary Doppler wire, CFR ≤2.5	Median follow-up of 12.5 years	Prevalence of CMD of 72% A trend toward worse mortality in endothelium-dependent CMD *vs*. preserved endothelial function Significantly worse mortality (adjusted HR 3.56, 95% CI 1.14–11.12, *p* = 0.03) in endothelium-independent CMD *vs*. CFR >2.5
Hage, 2020 [70]	n = 201 HFpEF, NA	TTE-derived CFR, CFR <2.5	Median follow-up of 388 days	Significantly higher rate of CV death or HF hospitalization in HFpEF patients with CMD *vs*. non-CMD
Kato, 2021 [71]	n = 163 HFpEF, NA	CMR-derived CFR, CFR <2.0	Median follow-up of 4.1 years	Significantly higher rate of all-cause death or HF hospitalization in HFpEF patients with CFR <2.0 *vs*. CFR ≥2.0
Rush, 2021 [57]	n = 106 HFpEF, NA	Endothelium-independent: CFR and IMR through coronary pressure wire, CFR <2.0 and/or IMR ≥25 Endothelium-dependent: abnormal coronary vasoreactivity after acetylcholine CMR-derived MPRI, MPRI ≤1.84	Median follow-up of 18 months	Prevalence of endothelium-independent CMD of 66%, endothelium-dependent CMD of 24%, and any CMD of 85% Prevalence of MPRI ≤1.84 of 71% (95% CI, 54–83%) No significant difference in clinical outcomes between patients with and without CMD Significantly higher rate of death or hospitalization (*p* = 0.011, log-rank) in patients with MPRI ≤1.84
Ozcan, 2021 [66]	n = 40 HFpEF, n = 40 non-HFpEF	CFR and IMR through coronary pressure wire, CFR <2.0, IMR >23	1 year	Trend toward higher prevalence of CMD in HFpEF *vs*. non-HFpEF patients (53% *vs*. 33%, *p* = 0.07) Significantly lower survival free of HF hospitalization in patients with abnormal CFR *vs*. those without
Mohammed, 2018 [69]	n = 22 HFpEF, n = 29 non-HFpEF	Endothelium-independent: CFR through coronary Doppler wire, CFR ≤2.5 Endothelium-dependent: increase in CBF after acetylcholine	NA	Significantly lower CFR (2.5 ± 0.6 *vs*. 3.2 ± 0.7; *p* = 0.0003) and median CBF increase (1 (−35; 34) *vs*. 64 (−4; 133); *p* = 0.002) in HFpEF *vs*. non-HFpEF patients Significant inverse correlations between CFR and PAWP at rest (r = −0.31; *p* = 0.03) and peak exercise (r = −0.47, *p* = 0.001)
Arnold, 2022 [72]	n = 101 HFpEF, n = 43 non-HFpEF	CMR-derived MPR, MPR <2.0	Median follow-up of 3.1 years	Significantly higher prevalence of CMD (70% *vs*. 48%, *p* = 0.014) in HFpEF *vs*. non-HFpEF patients In multivariable models, MPR was independently predictive of the composite of death or HF hospitalization in HFpEF patients
Mohammed, 2023 [68]	n = 137 HFpEF, NA	Pressure wire-free coronary angiography-derived IMR (caIMR), caIMR ≥25	Median follow-up of 15 months	Prevalence of CMD of 64.2% Using a multivariate analysis, CMD was independently predictive of all-cause death or HF hospitalization

Abbreviations: CFR, coronary flow reserve; CI, confidence interval; CMD, coronary microvascular dysfunction; CMR, cardiac magnetic resonance; CV, cardiovascular; HF, heart failure; HFpEF, heart failure with preserved ejection fraction; IMR, index of microcirculatory resistance; MBG, myocardial blush grade; MI, myocardial infarction; MPR, myocardial perfusion reserve; NA, not applicable; PET, positron emission tomography; Rb-82, rubidium 82; TFC, TIMI frame count; TTE, transthoracic Doppler echocardiography; TIMI, thrombolysis in myocardial infarction; CBF, coronary blood flow; MPRI, myocardial perfusione reserve index; PAWP, pulmonary arterial wedge pressure.

### 5.3 QOL

QOL refers to physical, psychological, emotional, and social features that are physiologically and socially constructed [73]. Assessing the QOL of an individual requires an extensive investigation: several functional tests and questionnaires have been implemented to explore all the QOL domains, including physical, mental, and social health.

Patients with CMD experience a worse QOL when compared to the general population, whereby all the QOL-related components are negatively affected.

#### 5.3.1 Physical Activity

The Duke Activity Status Index (DASI) questionnaire is the most adopted tool to assess the functional capacity of an individual and is a reliable surrogate of metabolic equivalents (METs) [74]. Several studies have documented poor physical status among patients with CMD. The landmark WISE registry [75] estimated a poor functional capacity (5.7 ± 4.2 METs) in a cohort of women with effort chest pain and non-obstructive CAD, while in the United Kingdom (UK)-Based INOCA International survey [14], the onset of symptoms was associated with a reduction in functional capacity of nearly 3 METs (5.6 ± 1.8 METs *vs*. 8.6 ± 1.8 METs, *p* < 0.0001), resulting in significant impairment of daily activities. These findings support the hypothesis that the angina burden of an individual represents the major determinant of physical status and that functional capacity progressively worsens with increased symptoms of chest pain.

Comparisons between patients with CMD and obstructive CAD in terms of physical performance are controversial. On the one hand, the WISE registry [76] and the UK-based INOCA International survey [14] reported a slightly greater functional capacity in the group with INOCA and CMD. Conversely, Schumann *et al*. [77] noted a close link between INOCA and worse physical performance, especially in the group with CMD. Accordingly, in the study by Jespersen *et al*. [78], the risk of long-term angina was higher in the groups with non-obstructive CAD (64%) and normal coronary arteries (49%) than in patients with obstructive CAD (41%).

The burden of persistent chest pain in the context of CMD is relevant and poses clinical and therapeutic concerns. Lamendola *et al*. [79], including 155 patients with cardiac syndrome X, reported that chest pain had remained unchanged in 33% and had worsened in 14% of patients at a mean follow-up of 137 ± 78 months. In addition, persistent chest pain often results in high rates of hospital readmission and repeated coronary angiographies with potentially harmful diagnostic procedures and a significant amount of costs for healthcare systems [14].

The assessment of functional capacity is of prognostic relevance. A functional capacity <5 METs, noted in 41% of patients with CMD [14], was an independent predictor of mortality in the WISE registry [75], while a reduction of every 1 MET in functional capacity resulted in a 3-day loss in physical health per month in the study by Gulati *et al*. [14].

#### 5.3.2 Mental Health 

Patients with CMD are likely to suffer from psychological disorders. Several psychometric questionnaires have been implemented to deepen the relationship between CMD and psychiatric comorbidities.

A landmark study by Potts *et al*. [80] documented that nearly two-thirds of patients with non-obstructive CAD experience psychological illnesses, including anxiety, panic disorder, and major depression. In the WISE registry, almost 45% of women suffered from depression [75], while Gulati *et al*. [14] reported impaired mental health and outlook on life in nearly 70% of patients, and up to 30% of subjects in small studies experienced panic disorder [81, 82]. An elegant investigation by Altintas *et al*. [83] established that anxiety disorder, depression, and somatoform diseases are the most prevalent psychological comorbidities in the context of CMD.

Pathogenesis of psychological disorders in CMD is multifaceted. First, the lack of awareness about CMD and the relatively low penetration of invasive functional tests in clinical practice often results in uncertainty concerning the source of chest pain [84] and delayed diagnosis. According to the UK-based INOCA International survey, half of patients receive a proper diagnosis of CMD more than one year after the onset of chest pain [14]. Second, patients with CMD are often burdened by nociceptive abnormalities and increased pain sensitivity that might fuel anginal complaints. Autonomic cardiac control imbalance might subtend interindividual differences in ischemic and pain thresholds [85]. Third, patients with CMD are frequently undertreated due to the false perception that CMD is a benign condition, potentially aggravating microvascular function and myocardial ischemia. Fourth, the high burden of anginal attacks with an unpredictable onset breeds a continuous state of apprehension that discourages patients from participating in social activities [84]. Fifth, the decline in physical status significantly worsens work performance with a detrimental economic impact, which further increases the risk of psychological disorders [86].

Convincing evidence showed that the severity and duration of chest pain correlate with the risk of psychological disorders. Pioneeristic studies documented an association between cardiac syndrome X and the risk of prolonged chest pain, panic disorder, and anxiety [75, 81]. Accordingly, a close link between the persistent chest and the burden of anxiety, depression, and psychotropic medication use was observed in the WISE registry [75].

Conversely, the burden of psychological abnormalities is not influenced by the extent of CAD and cardiac injury. Valkamo *et al*. [86] and van Schalkwijk *et al*. [87] found a similar rate of chest pain and mental disorders between patients with and without CAD. In addition, patients with CMD experienced significantly greater levels of anxiety compared to those with sudden cardiac death, suggesting that the rate of psychological illnesses is not linked to the nature of cardiac disease [77].

Importantly, psychological disorders can play a potential pathogenic role in CMD via enhanced sympathetic activity, low-grade inflammation, and endothelial dysfunction [85]. Consistently, an elegant study by Vermeltfoort *et al*. [88] demonstrated a positive association between trait anxiety and the extent of myocardial ischemia among 20 patients with CMD assessed using myocardial perfusion scintigraphy. In addition, psychological comorbidities facilitate the onset of unhealthy behaviors and classical cardiovascular risk factors, as well as enhanced platelet reactivity and procoagulant state, promoting the development of accelerated CAD [85]. Accordingly, Rutledge *et al*. [89] showed that depression and anxiety predicted the occurrence of MACEs among a cohort of 489 women with non-obstructive CAD at a median follow-up of 5.9 years.

#### 5.3.3 Social Activities

Up to 80% of patients with CMD state significant impairment in social activities, especially work performance [14]. After the onset of symptoms, nearly 75% of patients report missed days from work and fewer working hours per day. As outlined by the UK-based INOCA International survey [14], 47.5% retire earlier than expected from work, and nearly a third of patients are forced to change work due to significant physical impairment. Furthermore, physical, mental, and output limitations reduce work productivity [84]. These features culminate in significant economic consequences. Schumann *et al*. [77] estimated an annual economic impact of USD 21 billion due to INOCA-related premature exit from work, consistent with those of patients with obstructive CAD.

Finally, every 1 MET reduction in functional capacity led to 2.9 ± 0.7 days of inability to perform recreational activities [14]. All these findings increase the risk of psychological disorders [69] (Table 3, Ref. [14, 73, 77, 80, 82, 83, 84, 87]).

**Table 3.** **Most relevant studies focused on QOL in patients with CMD**.

First author, year [Ref]	Study population	Methodology	Baseline features	Main results
Potts, 1995 [80]	46 patients with NOCAD and 53 healthy controls	Standardized interviews and rating scales at the time of angiography, after 1 year and 11.4 years later	61% with psychiatric cases at angiography and 49% at 11.4 years	Levels of morbidity were significantly greater in patients with NOCAD Anxiety disorders were common, with panic disorder (15% of patients) the most common current diagnosis at final follow-up
Asbury, 2004 [82]	100 females with CSX, 100 females with CHD, 100 healthy female volunteers	HAQ questionnaire HADS scale	CSX patients were younger at the onset of symptoms (54 ± 8 years) than those with CHD (60 ± 9 years)	Syndrome X patients had higher levels of life interference (*p * < 0.05) and HADS anxiety (*p * < 0.05) than CHD patients, and higher levels of all HADS and HAQ scales than controls (*p * < 0.01)
Handberg, 2013 [73]	Sub-study of the WISE registry (936 patients with NOCAD)	BDI, STAI, DASI questionnaires Diamond chest pain questionnaire	Mean age 58 ± 12 years, 61% with NOCAD, 94% with discomfort	Persistent chest pain was associated with an increased rate of adverse events and relatively high rates of depression and anxiety with reduced functional capacity and impaired QOL over a median follow-up of 6 years
Altintas, 2014 [83]	56 CSX patients and 53 CHD patients	Structured clinical interview for DSM-IV axis I disorders BDI, BAI questionnaires Short form 36 scale	73.2% were women, 78.6% had a physical disease, 69.6% had experienced stressful life events	Depression was detected in 41% of the CSX group and 64% of the control group. Anxiety disorder was present in 64% of the CSX group. The somatoform disorder was determined in 24% of the CSX group
Schumann, 2021 [77]	66 patients with INOCA underwent stress cardiac magnetic resonance	QOL questionnaires (SAQ, CAQ, WLQ)	59% with definite or borderline CMD, 56.1% were women	INOCA patients reported: Lower SAQ scores, suggestive of worse symptoms than MI and stable CAD patients; higher CAQ scores, suggestive of greater cardiac anxiety than patients with sudden cardiac death; high WLQ scores, suggestive of significant work limitations
Gulati, 2023 [14]	297 patients from the United Kingdom-based INOCA International	Self-reported physical, social, and mental health. DASI score	60% with CMD, 91.2% were women	Functional capacity was poor after onset of symptoms (5.6 ± 1.8 METs) Adverse impact of symptoms on home life (80.5%), social life (80.1%), mental health (70.4%) In total, 75% of patients had reduced working activities, 47.5% retired early, and 38.4% applied for disability
van Schalkwijk, 2023 [87]	373 patients enrolled in the IMR and THIO studies	Modified SAQ Mental Health Continuum-Short Form Fatigue Assessment scale PHQ-9 General anxiety disorder questionnaire PSS	For INOCA: mean age of 62.74 years, 51% undertaking any alcohol use, and 69% being physically active	Compared to obstructive CAD patients, INOCA patients reported a better physical status lower smoking habits, obesity, and dyslipidemia, and similar rates of psychological distress and well-being
Humphreys, 2024 [84]	17 patients with INOCA	Qualitative investigation on lived experiences with INOCA	53% with CMD, 88.2% were women	Significant psychological impact on people living with INOCA Disappointment and lack of clarity over treatment and management pathways

Abbreviations: BDI, Beck depression inventory; CAD, coronary artery disease; CHD, coronary heart disease; CMD, coronary microvascular dysfunction; CSX, cardiac syndrome X; DASI, Duke Activity Status Index; HADS, Hospital Anxiety and Depression scale; HAQ, health anxiety questionnaire; INOCA, ischemia with no-obstructive coronary arteries; METs, metabolic equivalents; MI, myocardial infarction; NOCAD, non-obstructive coronary artery disease; PHQ-9, patient health questionnaire-9; PSS, perceived stress scale; QOL, quality of life; SAQ, Seattle Angina Questionnaire; STAI, Spielberger Trait Anxiety Inventory; WISE, Women’s Ischemia Syndrome Evaluation; WLQ, Work Limitations Questionnaire; CAQ, cognitive avoidance questionnaire; IMR, index of microvascular resistance; DSM-IV, Diagnostic and Statistical Manual of Mental Disorders, 4th edition; BAI, Beck anxiety inventory; THIO, the heart inside out.

## 6. Management of CMD

### 6.1 Available Therapeutic Options

The heterogeneous pathophysiology underpinning CMD and the need for more pertinent randomized trials pose concerns about the optimal management of CMD [90]. Beta-blockers, renin–angiotensin system inhibitors, and statins are the cornerstone CMD therapies [5].

Beta-blockers exert vasodilatory properties and are the mainstay therapy for CMD without evidence of coronary artery spasm. Nebivolol might be the most effective drug in the context of CMD and has been proven in an elegant study enclosing 38 patients with cardiac syndrome X to mitigate the frequency of angina attacks while improving functional capacity compared to metoprolol [91].

Renin–angiotensin system inhibitors participate in several vascular protection and vasodilatory pathways. The addition of quinapril on top of optimal medical therapy was able to improve CFR and lower the rate of chest pain in a sub-analysis of the WISE registry involving 78 women with CMD [92].

Statins prevent endothelial dysfunction via downregulation of ROS production and inflammatory pathways. In an elegant study by Fábián *et al*. [93], the administration of simvastatin resulted in a 52% relative increase in brachial artery flow-mediated dilation compared to the placebo.

### 6.2 Novel Therapeutic Strategies

Recently, novel therapeutic strategies have been demonstrated to mitigate angina symptoms and/or to improve microvascular function.

#### 6.2.1 ET-1 Inhibitors

ET-1 is a fascinating therapeutic target implicated in coronary vasoconstriction and endothelial dysfunction. Small clinical trials found improved myocardial perfusion after administration of ET-1 receptor antagonists [94, 95]. The large ongoing Precision Medicine with Zibotentan in Microvascular Angina (PRIZE) trial (ClinicalTrials.gov Identifier: NCT04097314) will address the potential role of zibotentan in improving functional capacity, assessed through treadmill exercise time, in a cohort of 356 patients with CMD.

#### 6.2.2 Coronary Sinus Reducer

Coronary sinus reducer implantation consists of the percutaneous deployment of a stainless-steel mesh in the coronary sinus. In recent studies, the putative ability to foster homogenous myocardial perfusion has been linked to high rates of angina relief and improvement in microcirculatory function [96]. The Coronary Sinus Reducer Objective Impact on Symptoms, MRI Ischaemia and Microvascular Resistance (ORBITA-COSMIC) trial enrolled 61 patients with CCS, angina, and evidence of myocardial ischemia unsuitable for coronary revascularization. At 6 months, coronary sinus reducer implantation reduced the number of daily angina episodes, with no evidence of improved myocardial perfusion [97]. In the INROAD (Index of Microcirculation Resistance Evaluation in Patients with Coronary Sinus Reducer Implantation) study, 21 patients with a history of coronary revascularization and refractory angina deemed not amenable for further revascularization underwent serial invasive coronary physiological assessment at 4-months. Here, the coronary sinus reducer lowered the rate of abnormal IMR by three times while significantly improving the CFR value and Seattle angina questionnaire summary score [98]. The ongoing COSIRA-2 (Efficacy of the Coronary Sinus Reducer in Patients With Refractory Angina II) phase 3 trial (Clinical Trial.gov Identifier: NCT05102019) will address the role of the coronary sinus reducer in patients with CMD.

#### 6.2.3 Autologous CD34+ Stem Cell Therapy

Evidence of defective endothelial cell function has provided the foundation for testing regenerative therapy in CMD using CD34+ stem cells. Preclinical studies outlined the ability of CD34+ cells to drive endothelial proliferation and microvascular repair. The ESCaPE-CMD (Autologous CD34 cell therapy for the treatment of coronary microvascular dysfunction in patients with angina and nonobstructive coronary arteries) [99] and the IMPROvE-CED (Intracoronary CD34+ Cell Therapy for Treatment of Coronary Endothelial Dysfunction in Patients with Angina and Nonobstructive Coronary Arteries) [100] trials showed a significant reduction in angina episodes and need for nitrate therapy as well as an increase in CFR after intracoronary infusion of CD34+ stem cells. The ongoing FREEDOM (Clinical Trial.gov Identifier: NCT04614467) placebo-controlled trial will further clarify the role of CD34+ stem cells in patients with CMD and persistent angina.

#### 6.2.4 Cardiac Rehabilitation Programs

The implementation of cardiac rehabilitation programs, when added to optimal medical therapy, has significantly benefited the physical and mental health of CMD patients. Leaf *et al*. [101] observed that a 12-week exercise training program improved functional capacity and ischemic ST changes during cardiopulmonary exercise training in patients with cardiac syndrome X. Later, a pivotal study by Eriksson *et al*. [102] showed additional benefits of physical training on endothelial function, neuroendocrine profile as well as anginal burden and QOL. Moreover, exercise training has recently been associated with improved CFV [103]. Indeed, high-intensity interval training (HIIT) has notably emerged as an effective strategy, showing superior benefits in exercise capacity, VO_2_ peak, endothelial function, cardiac function, and QOL compared to conventional moderate-intensity continuous training [104]. HIIT involves intermittent bouts of vigorous activity alternating with periods of active recovery and should be tailored to the functional capacity of each individual. Robust evidence supports the beneficial effects of HIIT protocols in patients with CAD [104, 105]. Interestingly, a pilot study investigated the potential benefits of an aerobic HIIT program consisting of treadmill exercises in a 4-minute × 4-minute format three times per week in patients with INOCA. After three months, the authors reported significant improvements in CFR, flow-mediated vasodilation, and VO_2_ max [106].

#### 6.2.5 Cognitive-Behavioral Therapy 

Cognitive-behavioral therapy is an emerging strategy for managing CMD, comprising a wide spectrum of interventions focused on stress control, optimized coping skills, relaxation techniques, and counseling programs. Cunningham *et al*. [107] found a significant improvement in terms of the burden of psychological disorders as well as physical status, anginal attacks, and exercise tolerance after a 12-week behavioral therapy in a cohort of nine women with cardiac syndrome X. The ongoing SAMCRO (Standardizing the Management of patients with Coronary Microvascular Dysfunction) study will investigate whether a multi-domain lifestyle intervention, consisting of dietary counseling, strict management of CV risk factors, tailored medical therapy based on the invasive assessment of CMD and coronary vasomotion, exercise training and psychological intervention, could help in improving individual’s QOL and rates of psychological disorders in a cohort of 120 patients with angina and non-obstructive CAD.

## 7. Creation of a Targeted Therapy for CMD 

The advent of invasive functional tests has delineated two major endotypes (structural and functional) of CMD, which largely differ in pathophysiology and invasive physiological characteristics [3]. Abnormal microvascular resistance is the key feature of structural CMD, while an imbalance between vasodilatory and vasoconstrictive agents has been postulated in functional CMD [32]. Whether these endotypes could benefit from targeted therapy has never been investigated. The ongoing MINOSSE (platelet and endothelial activation in angina with non-obstructive coronary artery disease and microvascular dysfunction) study will explore potential differences in platelet function and biochemical microenvironment between structural *vs*. functional CMD endotypes. This study could help identify novel potential therapeutic targets for CMD.

Finally, novel diagnostic and therapeutic strategies capable of forecasting the natural history of patients with CMD (the onset of CAD, vasomotor disorders, and/or HFpEF) and preventing adverse events are warranted.

## 8. Conclusions

CMD is a condition frequently encountered in clinical practice caused by a variable combination of structural and functional abnormalities of coronary microcirculation. CMD is associated with a high risk of complications, including MACEs and HFpEF, as well as a poor QOL. Evidence on novel therapeutic strategies, including cardiac rehabilitation programs, HIIT protocols, and cognitive–behavioral therapy, are largely awaited to improve the prognosis of patients with CMD.

## Abbreviations

CAD, coronary artery disease; CCS, chronic coronary syndrome; CFR, coronary flow reserve; CFV, coronary flow velocity; CMD, coronary microvascular dysfunction; CMR, cardiac magnetic resonance; DASI, Duke Activity Status Index; ET-1, endothelin-1; HIIT, high-intensity interval training; hMR, hyperemic microvascular resistance; HFpEF, heart failure with preserved ejection fraction; IMR, index of microcirculatory resistance; INOCA, ischemia with non-obstructive coronary artery disease; MACEs, major adverse cardiovascular events; MACCE, major adverse cardiac and cerebro vascular events; METs, metabolic equivalents; MI, myocardial infarction; MRR, microvascular resistance reserve; NO, nitric oxide; QOL, quality of life; PET, positron emission tomography; ROS, reactive oxygen species.
